# The Role of Interferons in Driving Susceptibility to Asthma Following Bronchiolitis: Controversies and Research Gaps

**DOI:** 10.3389/fimmu.2021.761660

**Published:** 2021-12-03

**Authors:** Heidi Makrinioti, Andrew Bush, James Gern, Sebastian Lennox Johnston, Nikolaos Papadopoulos, Wojciech Feleszko, Carlos A. Camargo, Kohei Hasegawa, Tuomas Jartti

**Affiliations:** ^1^ West Middlesex University Hospital, Chelsea, and Westminster Foundation Trust, London, United Kingdom; ^2^ Imperial Centre for Paediatrics and Child Health, Imperial College, London, United Kingdom; ^3^ National Heart and Lung Institute, Imperial College, London, United Kingdom; ^4^ Department of Paediatrics, School of Medicine and Public Health, University of Wisconsin-Madison, Wisconsin, WI, United States; ^5^ Allergy Department, 2nd Paediatric Clinic, National and Kapodistrian University of Athens, Athens, Greece; ^6^ Division of Infection, Immunity and Respiratory Medicine, School of Biological Sciences, The University of Manchester, Manchester, United Kingdom; ^7^ Department of Paediatric Pneumology and Allergy, The Medical University of Warsaw, Warsaw, Poland; ^8^ Department of Emergency Medicine, Massachusetts General Hospital, Harvard Medical School, Boston, MA, United States; ^9^ Department of Paediatrics, Turku University Hospital and Turku University, Turku, Finland; ^10^ Research Unit for Pediatrics, Pediatric Neurology, Pediatric Surgery, Child Psychiatry, Dermatology, Clinical Genetics, Obstetrics and Gynecology, Otorhinolaryngology and Ophthalmology (PEDEGO), Medical Research Center, University of Oulu, Oulu, Finland; ^11^ Department of Pediatrics and Adolescent Medicine, Oulu University Hospital, Oulu, Finland

**Keywords:** interferon, bronchiolitis, asthma, recurrent wheeze, rhinovirus, respiratory syncytal virus

## Abstract

Bronchiolitis is the most common cause of hospitalization in infancy and is associated with a higher risk for the development of childhood asthma. However, not all children hospitalized with bronchiolitis will develop asthma. The mechanisms underlying asthma development following bronchiolitis hospitalization are complex. Immune responses to respiratory viruses may underlie both bronchiolitis severity and long-term sequela (such as asthma). Interferons (IFNs) are important components of innate immune responses to respiratory viruses and could influence both asthma development and asthma exacerbations. However, the nature of the relationship between interferon production and wheezing illnesses is controversial. For example, low peripheral blood IFN responses at birth have been linked with recurrent wheeze and asthma development. In contrast, there is evidence that severe illnesses (e.g., hospitalization for bronchiolitis) are associated with increased IFN responses during acute infection (bronchiolitis hospitalization) and a higher risk for subsequent asthma diagnosis. Furthermore, mechanistic studies suggest that bronchial epithelial cells from asthmatic children have impaired IFN responses to respiratory viruses, which may enable increased viral replication followed by exaggerated secondary IFN responses. This review aims to discuss controversies around the role of IFNs as drivers of susceptibility to asthma development following bronchiolitis hospitalization. Past evidence from both mechanistic and cohort studies are discussed. We will highlight knowledge gaps that can inform future research study design.

## Introduction

Bronchiolitis remains the most common lower respiratory tract infection in infancy (1, 2). Cohort studies have shown that infants hospitalized with bronchiolitis in infancy have an increased risk to develop recurrent wheeze and asthma (3–6). It is of note though that most such studies defined asthma by medications’ use or by lung function tests and did not assess link to type 2 asthma phenotype (7). The relationship between bronchiolitis and asthma is complex and has been considered to be mediated by genetic, epigenetic, and environmental factors (8). Several reports have associated specific viruses, such as respiratory syncytial virus (RSV) and rhinovirus (RV) with an increased risk of recurrent wheeze and asthma diagnosis (6, 9). However, these associations may not be causal; they are potentially mediated by other factors (such as genetic predisposition and host immune responses) (10).

Interferons (IFNs) play a significant role in mediating early antiviral responses (11). The induction of type I IFNs plays a critical role in RSV-bronchiolitis. Genetically modified mice that do not express IFN-α/β and IFN-induced signalling mediators [(mitochondrial antiviral signalling (MAVS) or retinoic acid-inducible gene-I (RIG-I)] cannot exhibit increased viral replication (12, 13). The data around the role of IFNs in defining susceptibility to asthma following bronchiolitis are contradictory (14). Although low IFN levels at healthy status (birth) have been associated with recurrent wheeze and asthma, high IFN responses during acute disease are linked to asthma development (**Table 1**). The crux is that studies reporting associations between peripheral blood IFN levels and risk of either recurrent wheeze or asthma are based in measurements in peripheral blood samples that may not accurately reflect lung pathology (24). More recent studies have focused on analysing IFNs in upper airway samples (16). Possible sites of sample acquisition, the timing from the beginning of symptoms and associations with proinflammatory mediators have been considered to play an important role when assessing IFN responses in bronchiolitis (14).

**Table 1:** Interferon (IFN) types and their reported role in driving susceptibility to asthma following infant bronchiolitis.

IFN type	IFN receptors	IFN downstream pathway	Cohort studies	Mechanistic studies
Type I IFNs	IFN-α/-β receptor (**IFNAR1** and **IFNAR2** chains)	1. RLRs pathway (RNA viruses) viruses 2. cGAS-STING pathway (DNA viruses) 3. TRIF pathway (TLR3 and TLR4) 4. IRF7 pathway (TLR7, TLR8 and TLR9)	**a. Healthy (birth)** Low **cord blood type I IFN responses** associated with recurrent wheeze during 5 years of life (15) **b. Acute disease (severe bronchiolitis)** High **IFN-α transcript levels** in nasopharyngeal aspirates of infants with RSV-bronchiolitis associated with asthma diagnosis at 5 years old (16)	a. RSV is a weak inducer **of type I IFN expression** in plasmacytoid dendritic cells (p DCs) of neonatal mice (17) b. Induction **of type I IFN expression increasing with age** in mice (17) c. **Deficient type I IFN responses** following RV stimulation of bronchial epithelial cells from children with severe corticosteroid-resistant asthma (18) d. **Type I IFN responses** are elevated during RV-induced exacerbations of asthma in children with asthma (19)
(**IFN-α, IFN-β**, IFN-ϵ, IFN-κ and IFN-ω)		Type I IFNs phosphorylate STAT1 and STAT2 proteins through TYK2 and JAK1 STAT1 and STAT2 become dimer and associate with IRF9 and activate ISGF3 complex (activated transcription in an IFN-α dependent manner)		


Type II IFNs (**IFN-γ**)	IFN-gamma receptor (**IFNGR1** and **IFNGR2** chains)	1. IFN-GR1 subunit - associated with Jak1 2. IFN-GR2 subunit associated with Jak2 3. activation of Jak1 and Jak2 phosphorylation of STAT1 4. STAT1 phosphorylation homodimerization and nuclear translocation 5. STAT1 homodimers bind to IFN-gamma-activated sequence (GAS) elements in the promoters of target genes to regulate their transcription 6. IFN-gamma activates MAPK, PI3-K-Akt, and NF-kB signalling pathways to regulate the expression of a number of other genes	**a. Healthy (birth)** Absent **cord blood IFN-γ responses to viruses at birth** were associated with a higher incidence of acute wheeze during the first year of life (20)	**Upregulated peripheral blood IFN-γ secretion** upon “*ex vivo*” RV stimulation in children with asthma (19)
			**b. Acute disease (severe bronchiolitis)**	
			b1. Low **peripheral blood IFN-γ levels** during bronchiolitis hospitalizations associated with recurrent wheeze during first 2 years of life (21)	
			b2. High **IFN- γ levels** in nasopharyngeal aspirates of infants with RSV-bronchiolitis associated with asthma diagnosis at 5 years old (16)	
			c. In infants with **low cord blood IFN-γ responses,** lower respiratory tract infections are associated with increased peripheral blood IFN-γ responses during first year of life (22)	
Type III IFNs	Receptor complex consisting of **IL10R2 and IFNLR1**	1. IFN-LR1 subunit - associated with Jak1 2. IL10R2 subunit associated with Jak2 3. Activation of Jak1 and Jak2 leads to phosphorylation of STAT1 4. STAT1 phosphorylation homodimerization and nuclear translocation 5. Binding to IRF9 and activation of ISRE	**Lower cord blood type III IFN responses** to “*ex vivo*” stimulation with RV and stimulants was associated with higher risk of developing recurrent lower respiratory tract infections and persistent wheeze during the first 5 years of life (15)	1. **IFNLR1 knock out mice** had increased viral replication, inflammation and host damage following respiratory viral infection (23) 2. **deficient type III IFN responses** following RV stimulation of bronchial epithelial cells from children with severe corticosteroid-resistant asthma (18)
(**IFN-λ**)				

It has become evident that bronchiolitis is a heterogeneous condition (25, 26). This heterogeneity might complicate the identification of possible effective treatments, as a treatment that might be effective in some subgroups of infants with bronchiolitis, might not work for others. Innate IFNs seem to play an important role in bronchiolitis pathogenesis, but also in defining risk for subsequent chronic airway disease (recurrent wheeze, asthma) (15, 20). Their reported role is heterogenous too. Therefore, it is important to have a comprehensive understanding of the existing evidence with the aim to highlight controversies and knowledge gaps. This narrative scoping review aims to identify gaps in our understanding around the reported role of IFNs as mediators of susceptibility to subsequent asthma following hospitalization for bronchiolitis. Following scoping of the literature, we present the synthesis of the evidence from both cohort and mechanistic studies.

## The Role of Interferons in Driving Bronchiolitis Severity

Whether there is an association between innate IFN responses and bronchiolitis severity is as yet unclear. It has been shown repeatedly that infants with bronchiolitis have increased type II IFN expression and secretion in nasal lavage or nasopharyngeal aspirates as compared to healthy infants (27, 28). Some studies suggested that the severity of bronchiolitis is related to the infecting virus independent of IFN responses (29, 30). Other studies showed that RSV and RV bronchiolitis induce specific type III IFN subtypes that correlate with disease severity (31). Selvaggi et al. showed that infants hospitalized with severe RSV-bronchiolitis had higher nasopharyngeal IFN-λ1 as compared to infants hospitalized with severe RV bronchiolitis. However, virus type did not influence the correlation between IFN levels and severity (31). In contrast, Laham et al. demonstrated that distinct IFN profiles were driven by specific respiratory viruses and not by differences in disease severity (32).

One consistent finding is that robust innate immune responses are associated with decreased bronchiolitis severity. Back in 2007, Semple et al. showed that low nasopharyngeal levels of IFN-γ are associated with increased disease severity (33). In his study, 60 of 197 infants hospitalized with RSV-bronchiolitis required oxygen and ventilatory support and more than two thirds of them had significantly lower IFN-γ nasopharyngeal levels as compared to infants who did not require oxygen or ventilatory support (33). A recent study integrating clinical, virus, and multi-omics data in infants hospitalized with RSV-bronchiolitis provided additional insights. This study demonstrated that the cluster of infants with decreased IFN transcription in nasopharyngeal aspirates had more severe disease (16). Interestingly, this cluster of RSV-positive infants had a history of increased antibiotics prescription since birth, possibly due to an increased frequency of infections.

Therefore, low upper airway type II and III IFN protein levels have been associated with increased bronchiolitis severity. Both type II and III IFN protein and transcript levels in nasopharyngeal samples differ in response to different viruses (34). Further studies with additional insight on the timing of acute infection are required to define the role of IFNs as predictors of bronchiolitis severity.

## Cohort Studies

Data from cohort studies have established links between IFN levels detected in either upper airway or peripheral blood samples in infants with bronchiolitis and development of wheeze or childhood asthma. We discuss these findings in chronological order below.

In 1997, Renzi et al. followed up 26 infants hospitalized with bronchiolitis for 5 months. At the 5-month follow-up period, polymorphonuclear blood cells (PBMCs) were stimulated with interleukin-2 (IL-2) “*ex vivo*” and IFN-γ secretion was measured. Although the sample size was small, infants who had additional wheeze episodes during the follow-up period had significantly lower levels of IFN-γ secretion than those who did not wheeze again (21). Interestingly, the same team reported their findings from follow-up of these infants until the age of 2 years old. The infants who were hospitalized with bronchiolitis and required acute wheeze medication during the first 2 years of life had significantly lower PBMCs IFN-γ responses on admission to hospital and at 5 months follow-up when compared with infants who did not require acute wheeze medication (35). Also, the ratio of peripheral blood IFN-γ to IL4 was lower in infants who required medication for acute wheeze during follow-up.

**Figure 1:**
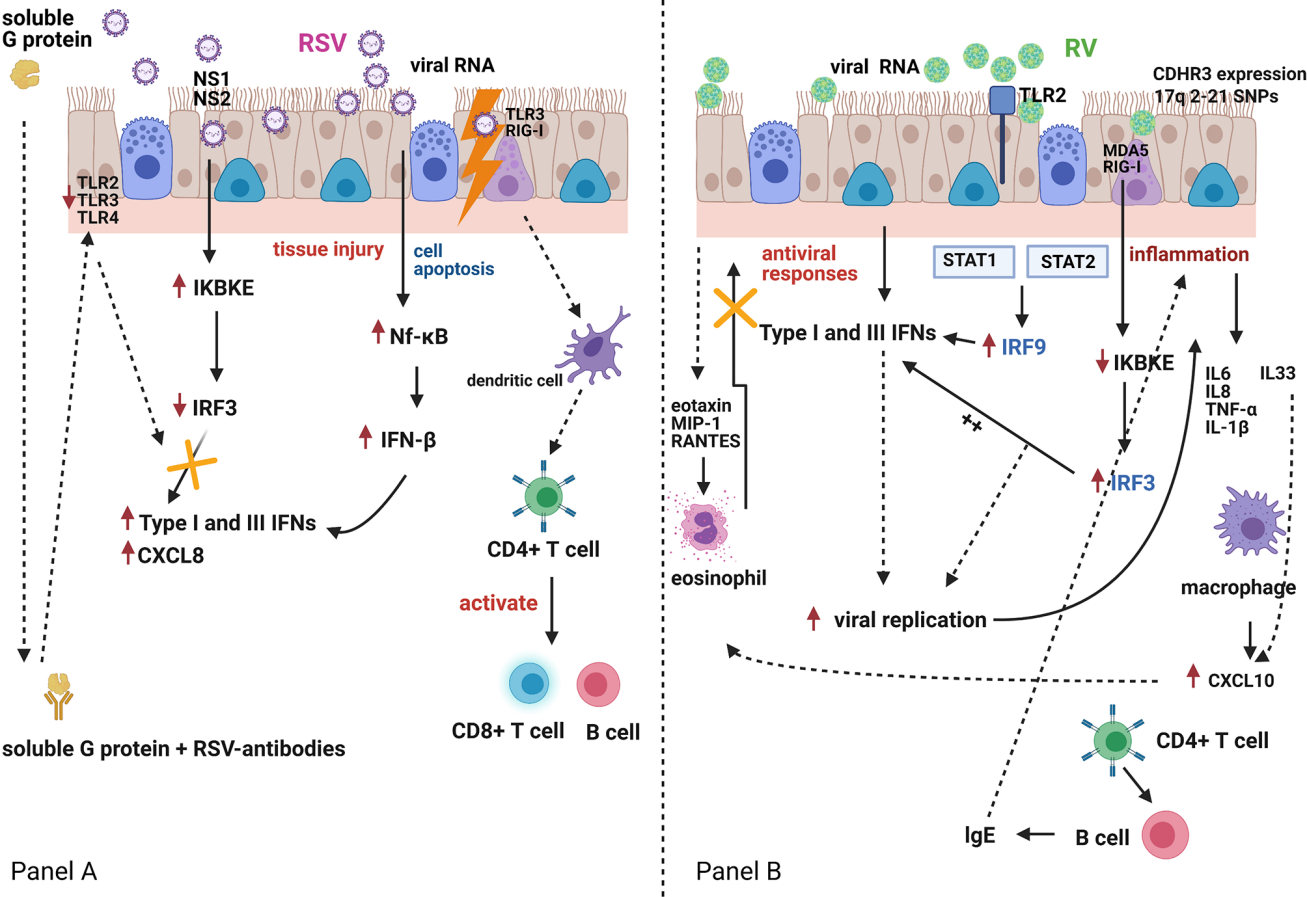
Respiratory Syncytial Virus (RSV) and rhinovirus (RV) infection and the host interferon (IFN)-mediated immune responses Panel **(A)**. RSV is bound by Toll-like receptor (TLR)-3 and retinoic acid-inducible gene (RIG)-I-like receptors. RSV can also infect dendritic cells, which carry viral antigens to regional lymph nodes. Presentation of viral antigens to CD4+ T-lymphocytes occurs, and primed T-cells activate B-lymphocytes and CD8+ T-cells. These migrate back to the infected epithelium with further release of mediators and recruitment of additional inflammatory cells. RSV antigen recognition leads to nuclear factor-κB (Nf-κB) activation with production of interferon (IFN)-β, a type-I IFN, which in turn and *via* an autocrine mechanism enhances its own synthesis and initiates the production of IFN-α, CXCL8 and type-III IFN (IFN-λ) by airway epithelial cells and innate immune cells. Both RSV nucleoproteins (NS1 and NS2) and RSV G-protein can antagonise the host immune response to RSV infection. Panel **(B)**. RV-induced TLR2 signalling induces interferon regulatory factor-3 and -9 (IRF-3 and IRF-9) activation, which in turn induces IFN expression which suppresses viral replication. However, RV infection of bronchial epithelial cells induces a virus-specific cytopathic effect, which is associated with an increased inflammatory reaction. Pro-inflammatory mediators (IL6, IL8, TNF-α, IL-1β) and pro-Th2 mediators (IL33), that are directly secreted by bronchial epithelial cells, induce eosinophil activation, and compete with the IFN-mediated antiviral activities. This may further amplify RV-induced inflammatory responses.TLR2, Toll-like receptor 2; TLR3, Toll-like receptor 3; TLR4, Toll-like receptor 4; NS1, non-structural protein 1; NS2, non-structural protein 2; MDA5, melanoma differentiation-associated protein 5; RIG-I, retinoic acid-inducible gene I; CXCL8, C-X-C Motif Chemokine Ligand 8; IKBKE, Inhibitor Of Nuclear Factor Kappa B Kinase Subunit Epsilon; STAT1, Signal transducer and activator of transcription 1; STAT2, Signal transducer and activator of transcription 2; MIP-1, macrophage inflammatory protein-1; RANTES, chemokine ligand 5; CXCL10, C-X-C Motif Chemokine Liga.

By contrast, Van Schaik et al. showed that infants who presented with acute bronchiolitis or with acute wheeze had higher peripheral blood IFN-γ to IL-4 ratios than healthy infants (36). This study included both infants with bronchiolitis and also recurrent wheeze and did not follow-up these infants to assess the incidence of subsequent wheeze or asthma, making comparison with other studies more difficult. Bont et al. followed up 50 infants with RSV-bronchiolitis for one year after admission to hospital and found no correlation between peripheral blood IFN-γ levels and the subsequent incidence of acute wheeze (4). This study assessed whole blood (not PBMCs) IFN-γ responses to several stimulants. Interestingly, the IFN-γ responses to phytohemagglutinin (PHA) in infants of atopic parents, were reduced as compared to other stimulants. PHA is a selective T cell mitogen (37). So, potentially these differences in IFN-γ responses could be attributed to the type of peripheral blood cells studied. Consistent with Renzi et al. evidence, Gern et al. reported in 2006 that undetectable RSV-induced IFN-γ cord blood mononuclear cell (CBMC) responses at birth were associated with a higher incidence of acute wheeze during the first year of life (20). Interestingly, RV-induced IFN-γ secretion were also significantly lower in infants who presented wheeze episodes during the first year of life as compared to those who did not present wheeze episodes. **Figure 1** summarizes schematically the reported differences in IFN regulation following RSV or RV infections of bronchial epithelial cells.

By contrast, Copenhaver et al. assessed cord blood IFN-γ levels in response to different stimulants and followed up infants for one year. Interestingly, infants with lower cord blood IFN-γ responses were more likely to present with recurrent upper and lower respiratory tract infections (including bronchiolitis) during the first year of life (22). Also, changes in IFN-γ responses were positively correlated with the number of illnesses during the first year of life. Some years later, Holt et al. expanded the findings of this study. In their study, Holt et al. followed up 151 infants at high risk to develop asthma (based on parental history of allergies and asthma) for 5 years. Cryopreserved CBMCs and PBMCs (age 4 years) were stimulated with polyinosinic-polycytidylic acid (poly [I:C]) and type I and type III IFN gene expression were measured. Infants with absent blood type I/III IFN responses had a higher risk for febrile lower respiratory tract infections and persistent wheeze during the first 5 years of life (15). In this study, the infants who experienced a febrile lower respiratory tract infection in the first year of life had restored their low type I/III IFN responses by age 4 years. Findings from these studies indicate that IFN responses at birth can influence the risk of acute respiratory illnesses, and that acute illnesses can promote development of IFN responses.

Assessing IFNs in upper airway samples has provided additional insights. In 2015, Nenna et al. showed that high type III IFN levels detected in nasopharyngeal washes of infants hospitalized with RSV-bronchiolitis correlated with an increased number of wheeze episodes during the first 3 years of life (38). Similar findings have been described for infants with parainfluenza-induced bronchiolitis (39). However, data from human metapneumovirus-positive infants with bronchiolitis showed that low IFN-γ/IL4 ratio in nasopharyngeal samples was associated with short-lasting post-viral wheeze (40). Hasegawa et al. integrated clinical, virological, and multi-omics data to identify endotypes of RSV-bronchiolitis and their associations with recurrent wheeze and asthma. Nasopharyngeal samples from infants recruited at the Multicenter Airway Research Collaboration-35 (MARC-35) cohort study were analysed for transcriptional responses to infection (16). The cluster of infants with RSV-bronchiolitis and background of paternal asthma and allergic sensitization, with high IFN-α and IFN-γ transcript levels in nasopharyngeal aspirates, were more likely to develop asthma at the age of five years. This cluster of infants with RSV-bronchiolitis were characterised by upregulated upper airway IFN-γ and Nf-κB pathways that likely induced an increase in type I IFN (IFN-β) transcription. Also, this cluster of infants had higher rate of recurrent wheeze that resulted in asthma diagnosis at the age of five years as compared to the cluster of infants with lower induction of type I/II IFN pathways in upper airway samples.

In summary, low peripheral blood type I and II IFN levels in baseline (not during acute disease) have been associated with increased incidence of recurrent wheeze and asthma through age five years. This association has not been vigorously confirmed in acute disease. In addition, data from upper airway samples show that in infants hospitalized with bronchiolitis, upregulated IFN transcription and increased type III IFN secretion during the acute infection is associated with higher risk for recurrent wheeze and asthma.

## Mechanistic Studies

Data from mechanistic studies have related innate IFN levels with airway hyperreactivity and remodelling. We discuss these data from “*in vivo*” and “*in vitro*” studies below.

The binding of type I IFNs to the IFN receptor (IFNAR) triggers the expression of numerous interferon-stimulated genes (ISGs) that have both antiviral and proinflammatory roles (17). Knockout mice for IFNAR had increased viral load levels and worse clinical outcomes (weight loss) during RSV infection (41). Similar outcomes were observed in IFN-α/-β knockout mice (23). Another study showed that RSV was a weak inducer of type I IFN expression in plasmacytoid dendritic cells (p DCs) of neonatal mice. The induction of type I IFN expression increased with age in these mice (23). This has not been observed in mouse RV models (42).

Mouse models have provided additional insights into the role of IFNs. Type III IFNs have reduced proinflammatory effects in comparison to type I IFNs and have anti-inflammatory and tissue-protective roles (43). IFNLR1 knock out mice had increased viral replication, inflammation and host damage following respiratory viral infection (43). Therefore, type III IFNs may limit initial viral replication without provoking unnecessary inflammation, while type I IFNs could represent a second line of defence to enhance antiviral responses by triggering proinflammatory responses. Airway inflammation, remodelling and hyperreactivity can be induced in mouse models following co-stimulation with viruses and allergens (house dust mite, cockroach extract) (18, 44). There is no mouse model of RSV or RV infection showing induction of “asthma” (as assessed by airway hyperreactivity and remodelling) by stimulation only with viruses. The synergistic play between allergen exposure and the respiratory virus in mice is mediated by impaired type I and III IFN induction and by increased secretion of type 2 cytokines and alarmins (particularly IL33) (18). Therefore, the balance between IFNs and type 2 mediators is important to determine chronic airway disease outcomes in “*in vivo*” models.

Impaired type I and III IFN responses have also been reported in “*ex vivo*” experiments with bronchial epithelial cells that are stimulated with respiratory viruses to model virus-induced asthma exacerbations. A study of bronchial epithelial cells from children with severe corticosteroid-resistant asthma reported reduced RV-induced type I and III IFN responses (19). Although type III IFNs were detected in supernatants from infected bronchial epithelial cells, IFN-β was only very mildly induced by RV stimulation. It was unclear whether this reflected impaired secretion due to intrinsic immune defect or secondary to asthma treatment or due to lack of sensitivity in the available testing methods for IFN-β. Deficient innate IFN responses were also reported in atopic non-asthmatic children, highlighting potential interactions between IFNs and type 2 immune responses (45). The role of timing of IFN responses during asthma exacerbations was assessed in a multicentre European study that showed that children with persistent exacerbations had deficient IFN-α responses at baseline, but these responses were restored during infection with respiratory viruses; RV was the most common virus isolated (46).

## Discussion

This narrative scoping review showed that impaired type I and III IFN expression and secretion in upper airway samples is associated with increased severity of viral respiratory illnesses. Both viral and individual factors likely contribute to IFN induction in severe bronchiolitis. Also, infants who develop severe bronchiolitis and have increased IFN transcription in upper airway samples during acute infection are more likely to develop asthma. On the contrary, impaired peripheral blood type I and II IFN expression prior to infection is associated with recurrent wheeze and asthma incidence. Thus, it is likely that the kinetics of the IFN responses may be critical factors, and that rapid induction of antiviral responses could limit viral replication and lead to better control of viral replication and less secondary induction of IFNs. Type I, II and III IFNs have some shared and distinct activities, and their specific roles in bronchiolitis pathogenesis is incompletely understood.

Allergic sensitization and the balance between IFNs and type 2 cytokine secretion also are associated with differences in the acute and chronic outcomes of viral respiratory infection. Impaired IFN-γ expression and secretion in upper airway samples has been associated with increased bronchiolitis severity (33). Kaneko et al. showed that infants with RSV-bronchiolitis and with evidence of allergic disease had significantly lower levels of IFN-γ in peripheral blood samples than infants with RSV-bronchiolitis and with no evidence of allergic disease (47). Kim et al. confirmed similar findings in bronchoalveolar (BAL) samples from infants with RSV-bronchiolitis (48). The presence of eosinophilia and high interleukin-5 levels in the BAL were more common in infants with hospitalized RSV-bronchiolitis in comparison to healthy infants. It is unclear whether pre-existing airway allergic sensitization impairs IFN responses to respiratory viruses during bronchiolitis or whether congenitally impaired IFN responses drive the inception of airway allergic sensitization and secondarily increase susceptibility to severe bronchiolitis (49).

Past studies showed that impaired type I and type III IFN expression and secretion in cord blood or peripheral blood samples at stable status (not acute infection) is associated with recurrent wheeze and asthma during the school years. It is of note that in these studies IFNs were measured in supernatants from stimulated peripheral blood cells. The secretion of type I and III IFNs in non-stimulated peripheral blood cells is minimal in these studies. Identifying IFN transcription pathways both during health and acute respiratory infection will help us understand whether what is described as “impaired IFN expression” is a result of physiological transcriptional regulation in different age groups or is an effect of timing of sampling in regard to infection progression. Data from the Manchester Birth Cohort Study offered a further perspective around the interactions between IFNs and other cytokine responses regarding the inception of asthma. The pattern of cytokine responses that was associated with troublesome early-onset asthma was characterised by diminished type I IFN responses to RV, and high proinflammatory cytokines (50). Therefore, the balance between peripheral blood IFNs and proinflammatory cytokines was shown to be predictive of asthma trajectories. A low ratio between type I IFNs and type 2 inflammatory responses (including pro-Th2 mediators) in upper airway samples has also been shown to be predictive of asthma exacerbations in school-age children with asthma (51).

By contrast, the presence of upregulated IFN transcription pathways in nasopharyngeal samples during a severe bronchiolitis hospitalization is associated with asthma at the age of 5 years. Raita et al. showed upregulation of IFN-α and IFN-γ transcripts (16). Type III IFNs (IFN-λ) are upregulated early during infection and the hospitalized infants were recruited during 4-5 days from beginning of symptoms. It would be interesting to see whether upregulation of interferon regulation factor (IRF) transcripts will correlate with severity and asthma outcomes. **Table 1** describes the main IFN types and their reported role in driving susceptibility to asthma inception following bronchiolitis.

## Gaps in Knowledge and Future Research Directions

Innate IFNs may play an important role in mediating pathways leading to the development of asthma following bronchiolitis in infancy (46). There are several knowledge gaps though, and future research can help clarify these. Main research gaps focus on the understanding of IFN kinetics following respiratory viral infection in children of different ages, on the use of IFNs as markers of bronchiolitis severity and/or markers of susceptibility to asthma. The timing of bronchiolitis infection, the virus type and proinflammatory mediators’ secretion can modulate the role of innate IFNs as links to asthma development, but it is still unclear how. We don’t know yet which are the pathways that underlie IFN expression in response to different respiratory viruses during severe bronchiolitis episodes. Mapping these pathways would help understand whether IFNs can be markers of bronchiolitis severity and can be used to identify groups of infants who will be benefited from early treatment. Also, future research should shed more light on interactions between upper and lower airway IFN transcription and the exposome (including air pollutants, allergens, microbes). More specifically, it will be interesting to define endotypes of bronchiolitis based on IFN transcription and post-transcriptional regulation and to assess the associations between these endotypes and asthma development. Nearly 7 out of 10 preschool children with wheeze will not be diagnosed with asthma (52). Could the regulation of IFNs play a role in this process? Then, targeted therapies in infants with severe bronchiolitis who are at risk to develop asthma could add significantly to asthma prevention. Lessons learnt from the use of omalizumab in children with atopic asthma highlight that careful endotyping can guide the development of most effective treatments (53). Future research can highlight whether IFNs could be used as treatment in severe bronchiolitis with potential disease modifying effect. Connecting methodological development (mathematical modelling) with biological data could drive problem solving in asthma development following bronchiolitis.

## Author Contributions

HM prepared the first draft of the manuscript, designed the figure and the table and made the editing following other authors’ comments. AB and JG worked reviewed the first draft of the manuscript, gave directions around the table and the figure design and worked on the editing of manuscript to help reach the final version. SJ, NP, and WF reviewed the manuscript. CC and KH reviewed the first draft of the manuscript and gave directions around the table and figure design. TJ invited HM to write the manuscript, was involved in the design of the manuscript map and reviewed and the first and final draft of the manuscript. All authors contributed to the article and approved the submitted version.

## Conflict of Interest

SLJ reports personal fees from Aquarius Bio, AstraZeneca, Bayer, Bioforce, BoehringerIngelheim, Enanta, GersonLehrman Group, Kaleido, LallemandPharma, Myelo Therapeutics ÐmbH, Novartis, resTORbio and Virtus Respiratory Research, outside the submitted work. In addition SLJ is an author on patents for the use of interferons for exacerbations of airway diseases. NP reports personal fees from Novartis, personal fees from Nutricia, personal fees from HAL, personal fees from MENARINI/FAES FARMA, personal fees from SANOFI, personal fees from MYLAN/MEDA, personal fees from BIOMAY, personal fees from AstraZeneca, personal fees from GSK, personal fees from MSD, personal fees from ASIT BIOTECH, personal fees from BoehringerIngelheim, grants from Gerolymatos International SA, grants from Capricare, outside the submitted work.

The remaining authors declare that the research was conducted in the absence of any commercial or financial relationships that could be construed as a potential conflict of interest.

## Publisher’s Note

All claims expressed in this article are solely those of the authors and do not necessarily represent those of their affiliated organizations, or those of the publisher, the editors and the reviewers. Any product that may be evaluated in this article, or claim that may be made by its manufacturer, is not guaranteed or endorsed by the publisher.
